# Quantification and dosimetric impact of intra‐fractional bladder changes during CBCT‐guided online adaptive radiotherapy for pelvic cancer treatments

**DOI:** 10.1002/acm2.70074

**Published:** 2025-03-21

**Authors:** Ingrid Valencia Lozano, Elizabeth Buss, Catherine S. Spina, David P. Horowitz, Lisa A. Kachnic, Michael Price, Yi‐Fang Wang, Reshma Munbodh

**Affiliations:** ^1^ Department of Radiation Oncology Columbia University Irving Medical Center New York New York USA; ^2^ Present address: Department of Medical Physics Memorial Sloan Kettering Cancer Center Montvale NJ USA; ^3^ Present address: Department of Radiation Oncology Saint Francis Hospital Hartford CT USA

**Keywords:** cone‐beam computed tomography (CBCT), intra‐fractional motion, online adaptive radiotherapy

## Abstract

**Purpose:**

This study quantitatively evaluates bladder changes and their dosimetric impact during the on‐couch adaptive process on a commercial CBCT‐based online adaptive radiotherapy (CT‐gART) platform.

**Methods:**

Data from 183 fractions of ten patients receiving online ART for pelvic cancers were analyzed retrospectively. Bladder contours were automatically generated and revised by an expert for each pair of planning and verification CBCTs. Bladder shape changes were assessed using geometric and boundary distance metrics. A deformable image registration (DIR) workflow was implemented to obtain spatial motion characteristics, validated by the dice similarity coefficient between bladder contours. Dosimetric parameters were quantified by warping ’intended’ dose distributions to the verification CBCT anatomy using DIR to evaluate coverage and OAR objectives.

**Results:**

Bladder volume changed noticeably during the on‐couch adaptation process (19.7 ± 3.3 min). Day‐to‐day bladder expansion showed an average increase of 3.4 cc/min ± 1.5 cc/min for the full bladder and 0.8 cc/min ± 0.3 cc/min for empty bladder protocols. Deformation occurred mainly in the superior region and was more pronounced for the full bladder protocol. Displacements over 5 mm in cranial‐caudal and anterior‐posterior directions averaged 16% and 5% of the volume for full bladders and 5% and 4% for empty bladders, respectively. CTV coverage (V100%) was maintained when the bladder was the target, but PTV V95% was reduced by an average of 7%. For non‐bladder treatments, bladder constraints increased slightly for supine subjects (0.5 Gy/fx), with prone subjects almost unaffected.

**Conclusions:**

A framework using auto‐segmentation and DIR was developed to evaluate the intra‐fractional motion of the bladder during CTgART. Results suggest that reducing the isotropic PTV margin to less than 7 mm may be feasible for oART, allowing patient‐specific anisotropic margins while maintaining the quality of the adaptive plan.

## 1 INTRODUCTION

Online adaptive radiation therapy (oART) aims to re‐optimize the plan while the patient is in the treatment position on the treatment couch, using imaging information to account for patient‐specific variations, including systematic and stochastic changes^1^, ^2^ These include anatomical changes such as tumor response, weight loss deformation, filling change, respiration, and peristaltic motion. The adaptation process seeks to optimize the therapeutic ratio by escalating or hypofractionating the target doses, while maintaining acceptably low toxicity to organs at risk (OARs).^3^

Cone‐beam computed tomography‐based online adaptive radiotherapy (CTgART) uses Cone‐beam CT (CBCT) imaging during a patient's treatment session to adapt the radiation delivery plan depending on the daily patient's anatomy. Fully integrated commercial solutions have become available in recent years^4^ (Ethos, Varian Medical Systems, Palo Alto, CA). This platform allows high‐quality images to be acquired using an iterative reconstruction algorithm.^5^ The system performs automatic segmentation based on daily patient anatomy by integrating advanced artificial intelligence tools and an automated plan re‐optimization workflow. Then, a preview of the dose distribution for the scheduled (original fluence) and adapted (new fluence) plans are generated.^6^, ^7^, ^8^, ^9^ At this point, the physician selects the better plan. Prior to the treatment, a CBCT image is acquired to verify the initial patient position and evaluate possible changes in anatomy, and the treatment plan is delivered to the patient.

Several authors have studied intra‐fractional bladder motion, particularly in non‐adaptive treatment setups.^10^, ^11^, ^12^ These studies indicate that motion can lead to three‐dimensional directional variations. However, these changes do not consistently correlate with the temporal trends or filling rates.^11^ Based on this previous research, the uncertainties related to intra‐fractional bladder changes are relatively small compared to the inter‐fraction variations. Hence, larger population‐based isotropic margins have been considered adequate to address this issue in the context of non‐adaptive radiotherapy,^13^ where the treatment duration is typically less than 10 min.

Studies in the field of online adaptive radiotherapy (oART) have concentrated on analyzing intra‐fractional volume variations and ensuring the repeatability of dosimetric constraints for MR‐guided adaptive treatments.^14^, ^15^, ^16^ It is important to note that larger margins, which are often used to compensate for inter‐fractional variations, may not be applicable to oART. This is because the adaptive workflow aims to address these variations.^17^ Therefore, to achieve further improvements in targeting accuracy and healthy tissue sparing, it is necessary to have a better understanding of the changes during the on‐couch adaptation process, and the need for proper dosimetric assessment. This information could then be exploited to optimize the margins based on the characteristics of CTgART treatments.

This study used a novel evaluation framework to assess the impact of intra‐fractional bladder variations during the on‐couch adaptation process in CTgART for pelvic cancer treatments. By means of AI auto‐segmentation and a state‐of‐the‐art diffeomorphic image registration algorithm (LDDMM),^18^, ^19^ we quantified changes in bladder shape and position and retrospectively assessed their dosimetric impact on a daily basis. Furthermore, the deformation vector fields (DVFs) generated by the DIR were used to determine the spatial characteristics of the bladder displacements. This methodology may offer a promising approach for characterizing on‐couch changes during CTgART and establishing patient‐specific anisotropic margins, while preserving the quality of the adaptive plan.

## 2 MATERIALS AND METHODS

Ten patients (six bladder, two prostate, one rectum, and one anal case) who received pelvic adaptive external beam radio‐therapy in the supine and prone positions were retrospectively analyzed under an institutionally approved protocol. A total of 183 fractions treated using the Varian ETHOS therapy system (Varian Medical Systems, Palo Alto, CA, USA) were analyzed. Before treatment for the full bladder filling protocol, subjects were instructed to drink an adequate amount of water, and a sufficient time interval was allowed between water intake and the session to ensure the stability of the bladder volume during the online adaptive procedure. The patients in the empty protocol were advised to void their bladder before each fraction of the treatment.

Each treatment fraction consisted of two sets of images. The pre‐treatment *planning* kV‐CBCT (pCBCT), which serves as the foundation for the adaptation process and is utilized for contouring and generating the synthetic CT for dose calculation.^20^ The second *verification* kV‐CBCT (vCBCT), which is acquired prior to dose delivery to confirm the final treatment position and accounts for any minor changes in the internal anatomy.^8^, ^21^ According to institutional guidelines, verification images were acquired using a fast protocol, resulting in reduced image quality.

The planning bladder contour corresponds to the contour generated by the ETHOS V1.1 (Varian Medical Systems, Palo Alto, CA) treatment management software and validated by the physician/adaptor. The verification images had no associated segmentation; therefore, a bladder contour was generated by the commercial AI auto‐segmentation software Autocontour (Radformation, New York, NY, USA) and reviewed and manually modified when needed by an expert radiation oncologist.

### 2.1 Contour‐based assessment

The pair of bladder contours obtained from the pCBCT and vCBCT underwent geometric analysis to quantify the volume variation during the on‐couch adaptation period. We calculated fractional changes and the corresponding expansion rate of the bladder, which was defined as the volume increase divided by the time between planning and verification CBCT, as indicated by the corresponding CBCT DICOM tag.^11^ Because volume‐based metrics provide limited clinical context and correlation with dosimetric quality,^22^ various authors recommend using distance‐based metrics that are more responsive to boundary inaccuracies.^17^ We calculated the Hausdorff distance (HD), defined as the maximum distance between the planning bladder contour and the nearest point on the verification contour on a 2D plane. In this study, we report the 95*^th^* percentile of HD (HD95%), as this value offers a suitable balance between sensitivity and specificity by mitigating the influence of outliers.

### 2.2 Longitudinal data and time trends

Statistical analysis of changes in bladder volume and HD was conducted based on the contours of all pCBCTs and vCBCTs. Differences within the patient cohort were quantified using group means and standard deviation (SD). Inter‐ and intra‐patients were calculated as the SD for each patient, respectively.

Linear regression was utilized to analyze the time trends, and a classical *t*‐test was employed to assess the statistical significance of the relationship between the on‐couch adaptation time and geometric variables.

### 2.3 Deformation‐based assessment

Structure‐guided DIR between pCBCT and vCBCT was performed to determine the spatial characteristics of bladder motion. The corresponding DVFs were computed using the large‐deformation diffeomorphic metric (LDDMM) with symmetric normalization (SyN) algorithm implemented in the open‐source software ANTS.^18^, ^23^ LDDMM is an image registration algorithm that models image transformation as a time‐dependent geodesic flow. This method guarantees that the deformation field is diffeomorphic, ensuring an invertible transformation between images while preserving the topology and anatomical features.

The registration parameters were modified from the standard parameters provided by ANTs developers to account for our specific pelvic CBCT‐to‐CBCT DIR problem. We performed an initial rigid alignment, and the DIR used mutual information as a similarity metric, a B‐spline transformation model, and a multi‐resolution approach with five levels. The DIR results were validated based on the dice similarity coefficient (DSC)^22^ as follows:

$$\mathrm{DSC}=\frac{2\left|A\cap B\right|}{\left|A\right|+\left|B\right|}$$

$$\left|A\right|+\left|B\right|$$

where *A* and *B* are the volumes of the deformed planning and verification bladders, respectively. The DVFs were mapped to the planning CT based on a second DIR.^24^ By doing so, the comparison of intra‐fractional variations in bladder anatomy is possible in common imaging space.

### 2.4 Dosimetric impact

To assess the impact of bladder intra‐fractional motion on the delivered dose distributions, planned and received doses were compared on a fractional basis. Since the verification CBCT does not provide accurate Hounsfield units for precise dose calculations, the dose distribution delivered to the anatomy of the verification image was obtained by warping the intended dose using the previously calculated DVFs for each fraction.^22^, ^25^ We generated and evaluated daily dose volume histograms (DVHs) for the *intended* (delivered plan as calculated on the anatomy of the pCBCT) and the *treated* (estimated dose on the anatomy of the vCBCT) dose distributions. The target coverage and OARs dosimetric planning constraint metrics were extracted, and the plans were compared based on the clinical dose objectives.

## 3 RESULTS

### 3.1 Geometric analysis and longitudinal data

The mean duration of each treatment course for the ten patients was 18 fractions, ranging from five to 28 fractions. The average duration of the on‐couch adaptation period was 19.7 min. Day‐to‐day variations in bladder filling were observed, with mean volume changes and expansion rates of 29.5% and 2.6 cc/min, respectively. The average bladder volume change for all subjects was 48.9 cc, with intra‐ and inter‐patient standard deviations of 24.4 and 43.8 cc, respectively. Additionally, HD95% exceeded 3 mm for all patients, with a maximum distance of 25 mm.

**FIGURE 1:**
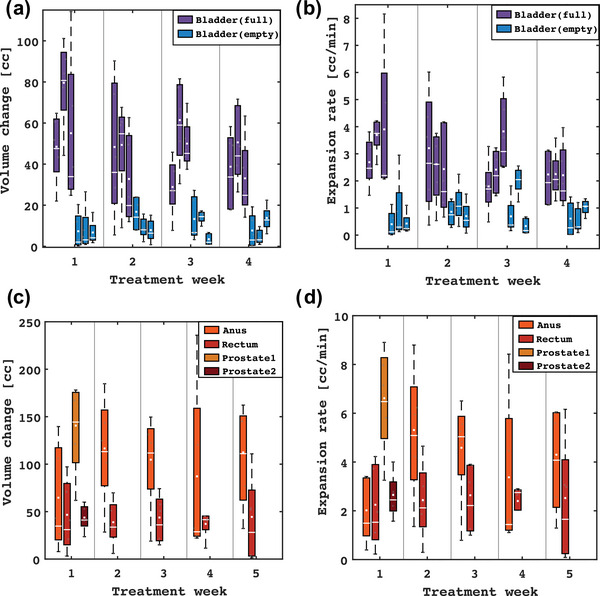
Box plots showing the weekly volume change (a and c) and expansion rate (b and d) for all subjects in the study for patients treated to the bladder and non‐bladder patients, respectively. Marked differences were observed between the full and empty bladders, with the weekly trends demonstrating greater consistency for the latter. The white central line and markers represent the median and mean values, respectively. The box depicts the range between the first and third quartile, and the whiskers extend from the box to show the data within 1.5 times the interquartile range.

Figure 1 shows the volumetric and geometric variations experienced by all subjects in the study, segregated into the bladder and other pelvic site cases. Bladder patients showed clear differences between the full and empty bladder groups (Figure 1a,b). Notably, the volume changes and expansion rates were substantially lower for the empty protocol, and the variations were more pronounced for the full bladders. The prostate subjects received hypofractionated treatments; hence, Figure 1c,d show data only from five days of treatment. Larger fluctuations were observed in all metrics for patients who were treated in the prone position (i.e., rectum and anus). Most patients who underwent treatment with a full bladder exhibited filling rates within a similar range (<7 cc/min). No discernible weekly trends were identified for the assessed metrics.

**FIGURE 2:**
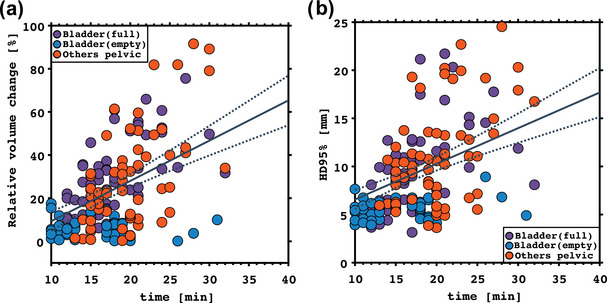
Scatter plots and regression analysis between relative volume change (a) and HD95% (b), and on‐couch adaptation time. In each plot, the solid line corresponds to the fitted curve, and dashed lines depict the confidence bounds of the linear regression model.

Figure 2 shows the trend of relative volume change and HD95% with time, which were analyzed using linear regression. Despite the strong positive correlation between the relative volume change and HD95% with on‐couch adaptive time (*p *< 0.001), there was considerable inter‐patient variability for both parameters, as evidenced by the coefficients of determination, which were *R* ^2 ^= 0.27 and *R* ^2^ = 0.20, respectively.

### 3.2 Intra‐fractional spatial variation

**FIGURE 3:**
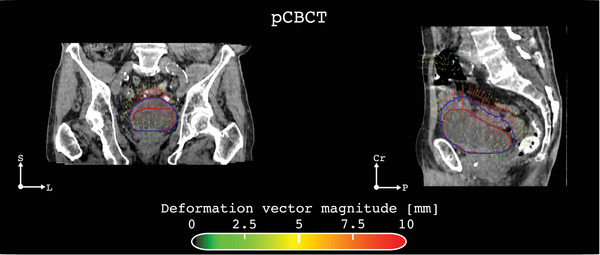
Case study patient for evaluating intra‐fractional bladder changes during CBCT‐guided online ART. Planning (red) and verification (blue) bladder contours are overlaid on the planning CBCT (pCBCT). The colored arrows indicate the direction and magnitude of anatomical changes during the on‐couch adaptation time. For this fraction, the time between pCBCT and vCBCT was 24 min.

**FIGURE 4:**
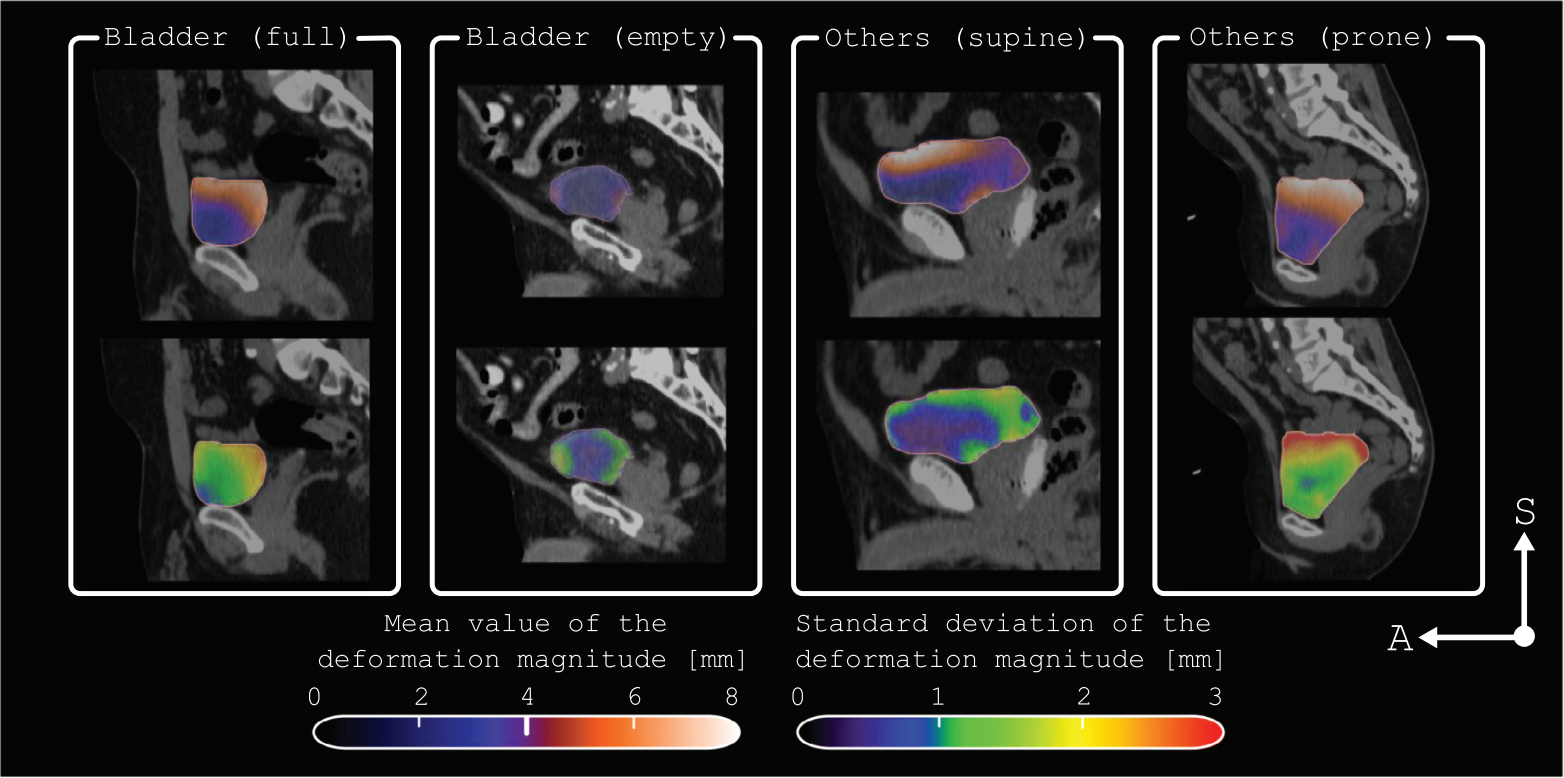
Averaged magnitude (upper row) and standard deviation (lower row) of the deformation vector fields projected on the bladder contour at the time of treatment simulation (CT SIM). Displacements in the superior region averaged more than 4 mm for all the subjects; however, the color maps also show the variability of bladder changes among the subjects in the study, contingent upon the filling protocol and treatment position.

For all 183 pairs of analyzed bladder contours, the overlap between the deformed planning bladder and the corresponding verification contour consistently yielded a high DSC (DSC > 0.9). This indicates robust agreement between the two segmentations, validating the reliability of the DIR. An example of a DVF for one session in one of the full bladder cases is shown in Figure 3. The predominant deformations were observed in the caudal‐cranial and posterior‐anterior directions, which matches the expected physiological changes in bladder volume and position due to filling and emptying. Moreover, local expansions of up to 100% (Jacobian determinant of 2) were primarily observed in the superior portion of the bladder. Figure 4 shows the spatial distribution of bladder deformation (mean and standard deviation over the treatment course) for four distinct subjects in our study. The mean displacement magnitude for full bladders was 4 mm (range [3.7–5.5 mm]), whereas the standard deviation reveals a substantial interpatient variation (range [0.7–2.7 mm]). In contrast, subjects with empty bladders demonstrated slightly reduced mean displacement values, averaging 3 mm. Notably, the standard deviation for empty bladders exhibited less variability.

Cumulative deformation histograms were computed to assess the bladder volume in relation to the magnitude of displacement. Table 1 summarizes the findings for displacements exceeding 3, 5, and 7 mm. These thresholds were chosen based on the institutional margins for non‐adaptive bladder treatments (1.0–1.2 cm) and the current adaptive margin of 7 mm. In general, the Cr‐Ca direction represented the largest displacement in most cases, with a maximum of 16% of the bladder experiencing displacements exceeding 5 mm. The L‐R direction demonstrated the smallest deformations in all the cases.

**TABLE 1:** Mean percentage of the bladder volume experiencing displacements larger than 3, 5, and 7 mm in each of the three cardinal directions by type of treatment for all ten subjects in the study.

		Bladder (full)	Bladder (empty)	Others (supine)	Others (prone)
Direction	Displacement (mm)	%			
Cr‐Ca	**>3**	34	14	30	32
	**>5**	16	5	13	12
	**>7**	7	2	5	4
A‐P	**>3**	20	16	23	18
	**>5**	5	4	10	5
	**>7**	6	2	5	2
L‐R	**>3**	12	4	23	15
	**>5**	2	1	5	4
	**>7**	0	0	0	0

### 3.3 Dosimetric evaluation

**FIGURE 5:**
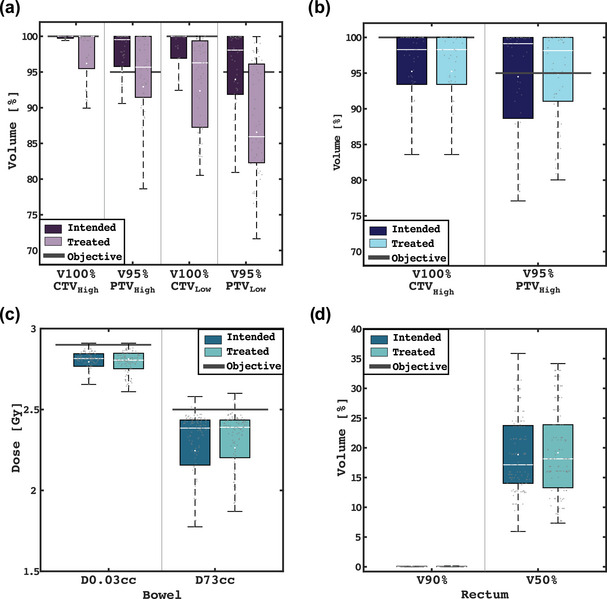
Dosimetric evaluation for the bladder treatment subjects. Box plots in (a) and (b) illustrate comparative intended and treated clinical target volume (CTV) and planning treatment volume (PTV) coverage for full and empty bladder protocols, respectively. Dose constraints for bowel and rectum are presented in (c) and (d) for all cases, irrespective of protocol.

*Bladder as target* DVH metrics of the treated (i.e., deformed) and intended fraction doses for bladder cancer patients are presented in the box plots in Figure 5. For the cases treated with a full bladder (using dose painting; Figure 5a), the median V100% was 96.1% and 92.9% for the high and low dose CTV, respectively. However, the PTV coverage was notably reduced. The PTV_high_ V95% median reached 86.6% (71.7%–100%), which was lower than the intended plan coverage of 94.0% (80.9%–100%), as well as the PTV_low_ V95% median was 92.4% (80.5%–100%), which was lower than the planned 100% (92.9%–100%) coverage. For cases treated with empty bladder (see Figure 5b), CTV and PTV coverage was consistent despite the changes during the adaptive session. Overall, the estimated doses to OARs were within institutional dose constraints with both protocols, as seen in Figure 5c,d.

*Bladder as OAR* For non‐bladder pelvic targets, the CTV (V100%) and PTV (V95%) coverage degradation in the delivered dose distribution was minimal, with an average decrease of less than 1%. The dose to relevant OARs, specifically the bowel (D0.03cc and D73cc) and rectum (V90% and V50%), also remained largely unchanged, with variations in relevant OAR metrics of less than 0.5 Gy. There was no significant difference between supine and prone positioning positions.

## 4 DISCUSSION

In this study, we assessed and quantified intra‐fractional variations in the volume, position, and shape of the bladder during the on‐couch adaptation time in CBCT‐guided oART for pelvic cancers. Previous research has documented that large bladder changes can occur throughout the course of radiation therapy, particularly in non‐adaptive settings.^11^, ^12^, ^26^, ^27^, ^28^ Our study proposes a framework that employs auto‐segmentation tools and DIR to assess the spatial changes that occur during the adaptive treatment process with a commercial CTgART platform. Additionally, we conducted a thorough evaluation of the impact of these changes on the intended dose distributions. The results revealed that although there was significant variation in bladder volume and expansion rate between patients, only 10% of the bladder volume underwent deformations greater than 5 mm in the cranial‐caudal direction. Anatomical changes did not result in considerable CTV coverage degradation, and, in most cases, the OAR objectives remain unaffected.

In our investigation, all subjects exhibited bladder changes during the around 20‐min on‐couch adaptation period, as evidenced by the range of HD95% values (12–25 mm). The differences in bladder volume and expansion rates among the 183 analyzed fractions were considerable, and there was a notable variation in the data when categorized on a weekly basis. This variability prevented us from identifying consistent behavior in individual bladder filling patterns, as none of these metrics emerged as reliable predictors for the rest of the treatment. The observed geometrical variations and substantial differences in volume changes and expansion rates are consistent with previous studies,^11^, ^26^, ^29^, ^30^ which similarly reported significant geometrical variations and differences in volume changes and expansion rates for the bladder.

The intra‐fractional relative volume change and dissimilarity of the pCBCT and vCBCT bladder contours (HD95%) increased with increased on‐couch adaptation time. In both cases, the correlation was relatively strong (*p *< 0.001), indicating a clear relationship between adaptation time and changes in bladder volume and shape.^31^ However, there was substantial interpatient variability (*R* ^2^ *∼* 0.27) that can be associated with different factors such as patient anatomy, physiological responses, or variations in fluid intake before treatment.^29^ Because the expansion rate changed over the course of the treatment, the relationship between the expansion rate and the initial volume was investigated. However, in line with previous studies,^11^, ^32^ no correlation was observed between these two variables. This supports that, during treatment, spatial changes in the bladder are influenced by factors beyond the initial filling state.

Our study's findings align with previous research, demonstrating that bladder displacements primarily occur in the cranial and anterior directions.^33^, ^34^ The results presented here, however, offer a unique insight within the CT‐guided oART, thereby providing a workflow that can be used to understand and account for inter‐fractional changes throughout the treatment. While MRgART enables real‐time tumor monitoring,^28^ CBCT adaptive approaches rely on implementing a margin to account for movement occurring during the workflow timeframe. For the analyzed fractions, small portions of the bladder (less than 13%) experienced displacements greater than 5 mm in these directions. This supports the implementation of anisotropic margins to account for the predominant asymmetrical motion due to bladder filling^35^ and potentially will be smaller than margins previously applied in patients with serial MRI scans, which ranged up to 14 mm cranially and anteriorly, 9 mm posteriorly, and 5 mm in all other directions.^32^ Moreover, adaptive workflows are intrinsically longer, which should be considered to minimize the negative dosimetric effects of bladder motion. Compared with MRgART 30–40 min sessions, CTgART treatments are shorter, and on average, an adaptive session can be completed in less than 20 min. At this time scale, an anisotropic margin ranging from 2 to 5 mm may be sufficient to account for intra‐fractional variations when the bladder is the target.^10^, ^32^, ^35^ Our limited study sample data suggest that there are unique patterns of bladder motion, and differences can exist among patients.^12^, ^36^ Consequently, tracking alone may be insufficient when the target also increases in overall volume.^21^ This finding emphasizes the need to implement anisotropic margins and underscores the importance of adaptive radiotherapy in the context of personalized medicine.

Regarding the differences between filling protocols, changes in volume and expansion rates were notably lower for empty bladders (see Figures 1 and 4). This pattern of changes aligns with previous studies that treat with empty bladder,^11^, ^37^, ^38^ which may enhance consistency and minimize potential variations during treatment. For these cases, OARs doses were only minimally affected across both empty and full bladder protocols, suggesting that using an empty protocol does not compromise surrounding normal tissue. The robustness of PTV coverage and dosimetric constraints, combined with the less pronounced changes observed with an empty bladder, suggests that CTgART treatments utilizing patient‐specific margins will be able to accommodate the range of motion likely to occur within the timeframe of the session. The potential for reduced intra‐fractional variations with empty bladder protocols may also simplify the adaptive planning process, potentially streamlining workflow and improving overall treatment efficiency.

Results of the dosimetric evaluation did not reveal any variation in CTV coverage despite bladder changes during the on‐couch adaptation time (see Figure 5a,b). This finding suggests that the margins currently considered in our practice are somewhat more conservative than the changes observed during the adaptive sessions. In cases where the bladder is an organ to protect, the metric that is most affected was D10% for subjects treated in the supine position (on average, increased by 0.5 Gy/fx). The dose increase may be attributed to a range of factors unique to this particular subset of patients. One possible explanation for this effect is the proximity of the bladder to the target area, which could result in variations in the high‐dose region when the patient is lying on their back. Additionally, the hypofractionated nature of the treatments leads to higher doses per fraction, which can accentuate the dosimetric impact of intra‐fractional bladder motion. Dosimetric assessment relied on the DVF from the DIR to estimate the treated dose distributions. This approach is a valuable tool for assessing doses during the course of treatment, and it has been employed previously in adaptive dosimetric studies.^32^, ^37^, ^39^ However, to define PTV margins, patient‐specific quality assurance of the DIR must be performed to ensure that uncertainties in the 3D dose distributions are considered.^40^ The level of uncertainty is higher in cases with significant anatomical changes. Our findings, along with the validation of the DIR using the DSC between both bladder contours, indicate that the uncertainty in our case was relatively small. Moving forward, obtaining the dose in the vCBCT may be possible using new imaging technologies that allow for dose calculation on CBCT images.^41^

While our findings offer a better understanding of the dynamics and dosimetric impact of bladder intra‐fractional motion, it is essential to acknowledge the limitations of our study, including the small sample size. Future research with a larger cohort of subjects and more extensive image data, encompassing diverse treatment positions and different bladder filling protocols, will facilitate the proposal of a margin recipe based on the patterns of per‐patient bladder changes. Such investigations will further validate the results presented herein and lead to a time‐efficient workflow and personalized margins to maintain the quality and advantages of adaptive treatments.

## 5 CONCLUSIONS

Intra‐fractional bladder changes during CBCT‐guided oART can be substantial, anisotropic, and correlated with adaptation time. Utilizing a methodology based on a commercial auto segmentation tool and DIR, it is possible to determine the spatial characteristics of bladder movement to quantify daily and average displacements. Furthermore, this approach allows for an assessment of the dosimetric consequences of motion between the planning and verification CBCTs without the inherent limitations of CBCT‐based dose calculations. Our findings offer a quantitative evaluation of the dynamic nature of bladder motion throughout the adaptive treatment process and emphasize the need for a time‐efficient treatment approach and the potential for developing personalized intra‐fraction margins to maximize the benefits of oART.

## AUTHOR CONTRIBUTIONS

Ingrid Valencia Lozano, Yi‐Fang Wang, and Reshma Munbodh: Conceptualization and methodology. Ingrid Valencia Lozano: Data curation, formal analysis, visualization, and writing – original draft preparation. Yi‐Fang Wang and Reshma Munbodh: Supervision and writing – review & editing. Elizabeth Buss, Catherine S. Spina, David P. Horowitz, Lisa A. Kachnic, and Michael Price: Resources and writing – review & editing. All authors discussed the results and approved the final version of the manuscript.

## CONFLICT OF INTEREST STATEMENT

The authors declare no conflicts of interest.
